# Lower Hemoglobin Following Adult Spine Deformity Surgery Leads to Longer Hospital Length of Stay

**DOI:** 10.7759/cureus.100340

**Published:** 2025-12-29

**Authors:** Justin Mathew, Jeffrey Gum, Steven D Glassman, Mladen Djurasovic, Morgan E Brown, Colleen Mahoney, Christy Daniels, Leah Carreon

**Affiliations:** 1 Orthopedics, Norton Leatherman Spine Center, Norton Healthcare, Louisville, USA; 2 Research, Norton Leatherman Spine Center, Norton Healthcare, Louisville, USA

**Keywords:** adult spinal deformity, blood transfusion, hospital length of stay, instrumented spine fusion, transfusion thresholds

## Abstract

Background

Patients commonly require blood transfusion for adult spinal deformity (ASD) surgery. Following the COVID-19 pandemic, the American Red Cross experienced the worst blood bank shortage in history, resulting in more rigid transfusion thresholds (RTT) across hospitals within the United States. This laboratory-driven threshold offers insight into the value of rigid criteria for transfusion utilization following ASD surgery.

Purpose

To evaluate the impact of RTT (Hemoglobin of 7.5g/dl) following ASD surgery on hospital length of stay (LOS) and to explore the relationship between nadir hemoglobin (Hgb) and LOS.

Methods

ASD patients from January 1, 2021 to June 30, 2021 (RTT) and from March 1, 2022 to August 31, 2022 Unrestricted Transfusion Threshold (UTT) were identified and compared with respect to demographic, operative, and postoperative outcomes including LOS.

Results

Fifty-one ASD patients were in the RTT group versus 43 in the UTT group. The two groups were similar in the number of females (61% vs 53%, p=0.533) and age (60.1 years vs 56.6 years, p=0.317). There was no difference in mean estimated blood loss (583.2ml vs 517.4ml, p=0.394) or operative time (291.9min vs 266.8min, p=0.187). Mean postoperative nadir (lowest) Hgb was similar between the cohorts as well (9.0 g/dl vs 9.3 g/dl, p=0.730). There was a higher percentage of RTT patients (8, 19%) with a nadir Hgb below 7.5 g/dl vs UTT patients (17, 33%), but there was no difference in the total number of patients reaching nadir values below 9.5, 8.5, or 7.5 g/dl. Across 94 patients, LOS was significantly longer at lower nadir hemoglobin, declining from 8.2 days (<7.5 g/dL) to 3.6 days (>9.5 g/dL) across ordered categories.

Conclusions

Rigid transfusion thresholds following ASD surgery did not impact nadir Hgb values nor LOS. Lower nadir postoperative Hgb values were associated with a longer LOS. Further investigation to understand this association could be beneficial for more objective transfusion recommendations with the potential to reduce LOS.

## Introduction

Adult spine deformity surgery (ASD) has potentially high volumes of blood loss [1] as it frequently involves long fusion procedures [2]. Postoperative spine patients generally experience postoperative anemia from intraoperative blood loss as well as blood loss in the postoperative period [3]. At many centers, hemoglobin is checked daily postoperatively to identify anemia and react accordingly with a variety of interventions including blood transfusion [4].

When transfusion is appropriate remains elusive, however. Many have advocated forestalling blood transfusion until hemoglobin falls below 7.0 [5,6]. However, the research around this threshold was generally developed in patients in the Intensive Care Unit (ICU) or from medical literature with chronic disease conditions. The Transfusion Requirements In Critical Care (TRICC) trial indicated that in critical care patients admitted in the ICU with hemoglobin <9 g/dL, more rigid transfusion thresholds did not adversely affect mortality rates [7]. In contrast, postoperative spine patients often start with more normal baseline hemoglobin levels, which drop relatively quickly, often predictably, following surgery. Moreover, many surgeons encourage aggressive mobilization as early as safely possible after surgery. However, with inadequate blood volumes and decreased tissue oxygenation associated with acute blood loss anemia, aggressive mobilization after surgery may not be possible. Therefore, as with surgical patients more generally, ASD patients benefit from aggressive steps towards early recovery.

The extent to which patients benefit from maintaining certain hemoglobin levels post-operatively remains controversial, with some studies suggesting higher levels to achieve an earlier recovery. In a study of hip fracture patients, maintaining a hemoglobin level above 10 g/dL did not increase mortality or impair the ability to walk postoperatively [6]. A more recent systematic review of adult spine surgeries suggests that postoperative hemoglobin should remain above 8 g/dL postoperatively and above 9 g/dL intraoperatively for adequate recovery [8].

The COVID-19 pandemic brought a period in which American Red Cross allogenic blood transfusion stores were low [9]. As a result, rigid criteria to transfuse patients were enacted for a period. This allowed our group to study the effects of a rigid transfusion threshold on ASD patients, in which patients could only be transfused for Hgb below 7.5 g/dL.

Prior to this study, it is unclear how restrictive transfusion thresholds affect patient outcomes, including nadir (lowest recorded value) hemoglobin and length of hospital stay after surgery. Moreover, studies of blood transfusion have not focused on ASD, a unique population of spine patients. This study, to our knowledge, is the first to assess postoperative length of stay (LOS) and outcomes between different transfusion thresholds among ASD patients.

The primary objective of this study is to determine if lower nadir hemoglobin is associated with longer hospitalization stays and if institution of Restricted Transfusion Thresholds (RTT) leads to lower transfusion rates and longer hospitalization stays.

This article was previously presented as a meeting abstract at the 2025 Spine Summit Meeting on February 23, 2025 and at the Scoliosis Research Society Annual Meeting on September 12, 2024.

## Materials and methods

This is a retrospective cohort study from a multi-surgeon single tertiary spine care institution. After receiving Institutional Review Board approval, the clinical records of 94 patients with ASD who underwent deformity correction and spinal fusion with instrumentation over more than five levels were reviewed. Patients who underwent surgery for a tumor, trauma, or infection were excluded. During the COVID-19 pandemic, when the blood supply was in shortage, we included 43 patients from January 1, 2021 to June 30, 2021. We label this as the restricted transfusion threshold (RTT). During this period, transfusion was deemed necessary if the patient's serum hemoglobin level was 7.5 g/dL or less. If the spine surgeon decided that transfusion was necessary in a patient with serum hemoglobin 7.5 g/dL or more, a non-spine physician needed to agree that the transfusion was necessary.

Following the pandemic, the RTT was lifted. From March 1, 2022 to August 31, 2022, 51 additional adult spine deformity patients were identified in the unrestricted transfusion threshold (UTT) period. Patient demographics, including age, sex, body mass index (BMI), American Society of Anesthesiologists Physical grade (ASA), smoking status, diagnosis of diabetes mellitus, and history of lumbar spine surgery, were collected. Surgical parameters were collected, including the number of surgical and interbody fusion levels, estimated blood loss (EBL), and operative time. Postoperatively, hemoglobin values, including nadir hemoglobin, and LOS were recorded.

Continuous variables were summarized as mean ± standard deviation (SD), and categorical variables as frequencies with percentages. The Shapiro-Wilk test was used to assess the normality of continuous variables. Unpaired independent t-tests were used to compare normally distributed continuous variables, and the Mann-Whitney test was used to compare non-normally distributed continuous variables between the RTT and UTT groups. Categorical variables were compared using Fisher’s exact test. Repeated measures ANOVA was performed to compare the trajectories of hemoglobin values over time, post-operative day 1 to post-operative day 6, between the two groups. Regression analysis was performed to identify factors associated with length of stay. Statistical significance was defined as p<0.05. All analyses were performed using SPSS v29.0 (IBM Corp., Armonk, NY, USA).

## Results

A total of 43 patients were observed in the RTT period. Fifty-one patients were observed in the UTT interval. Demographically, the groups were similar: there were no significant differences in age (56.6 vs 60.1 years old, p=0.317), BMI (29.3 vs 32.1, p=0.131), or pre-operative Hb levels (13.30 vs 12.83, p=0.201) (Table 1).

**Table 1:** Summary of demographic data and surgical parameters between the two cohorts *p-value from Fisher's exact test ^+^p-value from unpaired independent t-tests / Mann-Whitney test ^a^ U-statistic ^b^ t-statistic

	Restricted Transfusion Threshold	Unrestricted Transfusion Threshold	p-value	Test-statistic
N	43	51		
Sex, N (%)			0.533*	
Females	23	31		
Males	20	20		
Age, years, Mean (SD)	56.56 (17.77)	60.12 (16.21)	0.317^+^	1258.00^a^
BMI, kg/m^2^, Mean (SD)	29.27 (7.88)	32.07 (6.63)	0.131^+^	-1.305^b^
Estimated Blood Loss, cc, Mean (SD)	517.44 (316.14)	583.24 (426.97)	0.394^+^	1129.00^a^
Operative Time, min Mean (SD)	266.81 (80.81)	291.88 (101.49)	0.186^+^	-1.135^b^
Pre-operative Hb, Mean (SD)	13.30 (1.45)	12.83 (2.04)	0.201^+^	1.289^b^
Lowest Post-Op Hb, Mean (SD)	9.26 (1.50)	9.03 (2.07)	0.730^+^	0.513^a^
Lowest Hb Categorical			0.091*	
≤7.5	8	17		
>7.5 / ≤8.5	10	8		
>8.5 / ≤9.5	11	5		
≥9.5	14	21		
LOS, days, Mean (SD)	5.37 (3.71)	5.88 (4.55)	0.551^+^	566.50^a^

Likewise, intraoperative metrics were similar. EBL was comparable between the groups (517.4 vs 583.2 mL, p=0.394), as was operative time (266.81 vs 291.88 minutes, p=0.186). Postoperatively, there was no difference in mean nadir Hgb between the groups (9.3 vs 9.0, p=0.730). There was a lower percentage of patients that had a nadir Hgb < 7.5 g/dl in the RTT interval group (N=8, 19%) vs UTT (N=17, 33%). During the postoperative period, the mean hemoglobin level trended downwards through the third post-operative day (POD3) in both groups and continued to trend lower in the RTT group through the sixth post-operative day (POD6) (9.26 vs 8.83); but this did not reach statistical significance (p=0.074) (Table 2).

**Table 2:** Hemoglobin values (g/dL) Mean (SD) for each post-operative day (POD) *p-value from repeated measures ANOVA, F-statistic = 5.294

Post-operative day	Restricted Transfusion Threshold	Unrestricted Transfusion Threshold	p-value
			0.074*
POD 1	9.80 (1.40)	9.49 (1.82)	
POD 2	9.41 (1.36)	9.15 (1.75)	
POD 3	9.03 (1.31)	8.60 (1.38)	
POD 4	9.19 (1.25)	8.85 (1.05)	
POD 5	9.19 (1.34)	8.66 (1.15)	
POD 6	9.26 (1.33)	8.83 (0.72)	

Between groups, there were no significant differences (p=0.399) in the number of patients transfused for different hemoglobin levels (Table 3).

**Table 3:** Proportion of patients transfused stratified by Nadir during post-operative stay between the two cohorts ^+^p-value from Fisher's exact test

	Restricted Transfusion Threshold	Unrestricted Transfusion Threshold	p-value^+^
Nadir Hemoglobin, g/dL	N	N	0.399
≤7.5	5 of 8 (63%)	9 of 17 (53%)	
>7.5 / ≤8.5	0 of 10	0 of 8	
>8.5 / ≤9.5	1 of 11 (9%)	0 of 5	
≥9.5	0 of 14	0 of 21	

Mean LOS was not significantly different between the two groups (5.4 vs 5.9 days, p=0.551). However, when stratifying the patients by nadir hemoglobin values, there were significant differences seen in each group (Table 4).

**Table 4:** Mean length of hospital stay, days, Mean (SD) stratified by Nadir Hemoglobin during post-operative stay between the two cohorts *within cohort difference (ANOVA) **between cohort (Pre vs Post) difference t-statistic = -1.047

	Restricted Transfusion Threshold	Unrestricted Transfusion Threshold	p-value
Nadir Hemoglobin, g/dL			0.275**
≤7.5	7.75 (3.11)	8.35 (6.51)	
>7.5 / ≤8.5	6.50 (5.50)	6.75 (2.25)	
>8.5 / ≤9.5	5.36 (2.87)	4.40 (2.79)	
≥9.5	3.21 (1.58)	3.90 (2.1)	
p-value	0.024*	0.016*	
F-statistic	3.367	3.819	

Specifically, patients with lower nadir hemoglobin had significantly longer LOS in the hospital, compared to those with higher nadir hemoglobin values (p=0.024). This was consistent among both the UTT and RTT groups. Across 94 patients, LOS was significantly longer at lower nadir hemoglobin, declining from 8.2 days (<7.5 g/dL) to 3.6 days (>9.5 g/dL) across ordered categories (p=0.275, Table 4).

Regression analysis showed that only nadir Hb was associated with LOS (Table 5).

**Table 5:** Regression analysis with length of stay as dependent variable Hb: Hemoglobin, RTT: Restricted Transfusion Threshold, UTT: Unrestricted Transfusion Threshold

	Standardized β coefficient	t-score	p-value
Sex	0.236	1.792	0.078
Age	0.216	1.816	0.074
Body Mass Index	0.027	0.243	0.809
Estimated Blood Loss	-0.075	-0.563	0.575
Operative Time	0.315	2.218	0.030
Pre-operative Hb	0.017	0.142	0.887
Lowest Post-Op Hb	-0.408	-3.465	<0.001
Cohort (RTT vs UTT)	0.005	0.047	0.963

## Discussion

It has been postulated that transfusing patients above a certain hemoglobin level might be wasteful and potentially harmful. This is especially so when allogeneic blood is in limited supply. The FOCUS (Functional Outcomes in Cardiovascular Patients Undergoing Surgical Hip Fracture Repair) study indicated that hip fracture patients with preexisting cardiac disease and a postoperative Hgb <10 did not have better outcomes when transfused liberally, when Hgb <10, than those patients who were transfused more restrictively (with symptomatic anemia or hemoglobin <8 when recommended by the physician) [10]. Similarly, the TRACS (Transfusion Requirements after Cardiac Surgery) randomized controlled trial indicated that there was no inferiority when comparing 30-day mortality or severe morbidity [11]. Another study focusing on cardiac surgery patients observed that patients with postoperative transfusion often had adverse outcomes including a higher risk of infection [12]. Notably, much of the literature inveighing against less restrictive transfusion thresholds focuses on critically ill patients with underlying cardiac conditions. In contrast, there is a relative paucity of literature regarding transfusion thresholds in patients undergoing ASD correction. Many of these patients are not critically ill and are undergoing elective surgery with potentially higher volumes of blood loss.

There are a number of studies that do assess postoperative length of stay and suggest, similarly to our study, that postoperative hemoglobin levels may predict an increased length of stay. One study in hip fracture patients found nadir hemoglobin predicted readmissions and length of stay [13]. Another study examined patients undergoing lumbar spine fusion and observed that postoperative nadir hemoglobin was more predictive of LOS than hemoglobin on POD1 [14]. These conclusions are consistent with the results of our study. While our data did not address patient outcomes or mobilization in the ASD population, it did show that the RTT group seemed to have lower hemoglobin towards POD 6, though this did not reach significance in this study. It is possible that this difference may have become significant with a larger sample size.

Our results did not show a significant difference in LOS between the RTT and the UTT groups, even when stratifying by different nadir hemoglobin levels. Notably, the patients in the UTT were not transfused differently between different nadir hemoglobins, which would make it challenging to draw conclusions about differences in length of stay between the groups. However, within each group, patients with lower nadir hemoglobin had longer hospital LOS. As might be expected, among the RTT group, there was a greater proportion of patients with hemoglobin ≤7.5 (17 of 51 patients, 33%) than in the UTT (5 of 43 patients, 12%). Overall, no patients were transfused with hemoglobin over 7.5 except for one patient with hemoglobin <9.5. Our limited sample size, however, may affect our results: it is possible that with larger sample sizes, we may see a greater proportion of patients receiving transfusion with higher hemoglobin values, especially when there is a significant drop in hemoglobin from the preoperative baseline. Studies that prospectively study transfusions automatically triggered by different hemoglobin thresholds, or by drops in hemoglobin from baseline, in the ASD population will be useful to assess the effect of earlier transfusions on metrics such as LOS in this population of patients.

This study does have limitations. The retrospective nature of this study introduces some potential selection bias and confounding variables. Transfusion decisions may reflect unmeasured severity, intraoperative events, or clinician judgement, and LOS can be affected by discharge criteria, rehabilitation pathways, and social factors. Notably, we did assess for significant differences in patient characteristics in each group to account for this. Moreover, the limited sample size in this study reduces the power of the study, which may explain why many of our findings did not show statistical significance, though they appeared different. This is also a study from a single tertiary center, and practices may differ elsewhere. Determining the tipping point of transfusion benefit relative to cost per extra day LOS would be useful for both clinical uses and financial optimisation. However, this is difficult to assess in the current study due to the heterogenous nature of the cohort.

## Conclusions

Postoperative length of stay was not significantly different in ASD surgery with the use of rigid transfusion thresholds compared to those without such rigid thresholds. Moreover, nadir hemoglobin values did seem to be lower overall with the use of a rigid transfusion threshold, though this did not reach statistical significance. Patients in both groups whose nadir hemoglobin levels drifted to lower values did stay significantly longer in the hospital than those whose hemoglobin nadir was higher. Additional larger studies should examine whether more liberal transfusion thresholds can reduce length of stay by preventing inpatient hemoglobin values from falling below certain values.
